# Genetic epidemiology of moyamoya disease and CADASIL in over 120,000 healthy Korean individuals: Insights into cerebrovascular disorders

**DOI:** 10.1371/journal.pone.0331174

**Published:** 2025-09-22

**Authors:** Eun Hye Cho, Myoungkeun Lee, Chang-Seok Ki, Chang Ahn Seol, Mi-Ae Jang

**Affiliations:** 1 Department of Laboratory Medicine, Kangbuk Samsung Hospital, Sungkyunkwan University School of Medicine, Seoul, Republic of Korea; 2 GC Genome, Yongin, Republic of Korea; 3 GC Labs, Yongin, Republic of Korea; 4 Department of Laboratory Medicine and Genetics, Samsung Medical Center, Sungkyunkwan University School of Medicine, Seoul, Republic of Korea; University of Vermont, UNITED STATES OF AMERICA

## Abstract

**Background:**

East Asia has one of the highest global stroke burdens. Genetically, moyamoya disease (MMD) and cerebral autosomal dominant arteriopathy with subcortical infarcts and leukoencephalopathy (CADASIL) contribute to this burden. This study investigated the allele frequencies and estimated the genetic prevalence of *RNF213* and *NOTCH3* variants in a large Korean population.

**Methods:**

Between July 2021 and August 2024, 129,933 individuals who underwent health checkups were included. *RNF213* p.Arg4810Lys and three *NOTCH3* variants, p.Arg544Cys, p.Arg640Cys, and p.Arg75Pro, were analyzed using next-generation sequencing. Allele frequencies were calculated, and genetic prevalence was estimated using the Hardy-Weinberg equilibrium.

**Results:**

The allele frequency of *RNF213* p.Arg4810Lys was 1.08%, with 13 homozygotes. The estimated genetic prevalence of MMD was 1 in 47 individuals. For *NOTCH3*, the allele frequencies were 0.07% for p.Arg544Cys, 0.07% for p.Arg640Cys, and 0.04% for p.Arg75Pro, corresponding to an estimated genetic prevalence of CADASIL of 1 in 277 individuals. Eight individuals carried both the *RNF213* and *NOTCH3* variants.

**Conclusion:**

This large-scale study highlights the substantial genetic burden of *RNF213* and *NOTCH3* variants in Koreans, which partially contributes to the high stroke burden in East Asia. These findings emphasize the importance of population-specific genetic studies to optimize cerebrovascular disorder management in East Asia.

## Introduction

Stroke is the second leading cause of death and the third leading cause of death and disability among non-communicable disorders worldwide [1]. According to the Global Burden of Disease Study 2021, East Asia is one of the regions with the highest stroke burden [1]. This elevated risk is attributed to a combination of environmental, lifestyle, and genetic factors. Among the genetic contributors, moyamoya disease (MMD) and cerebral autosomal dominant arteriopathy with subcortical infarcts and leukoencephalopathy (CADASIL) play significant roles in the regional burden of cerebrovascular disorder. MMD and CADASIL are cerebrovascular disorders characterized by progressive vascular abnormalities that increase the risk of stroke [2,3]. Both conditions are prevalent in East Asia and are attributed to population-specific genetic variants and founder effects.

MMD is characterized by progressive stenosis of large intracranial arteries and subsequent development of fragile collateral vessels as a compensatory mechanism [2]. The inheritance pattern is generally considered autosomal dominant with incomplete penetrance [4]. However, early onset and severe cases have also been reported in homozygous individuals, suggesting that autosomal recessive inheritance may also play a role [5,6]. The prevalence of MMD differs significantly among ethnic groups. An epidemiological study in the United States reported that, compared to whites, the incidence rate ratios for MMD were 4.6 for Asian Americans, 2.2 for Black individuals, and 0.5 for Hispanics, highlighting a marked disparity in risk across populations [7]. This finding aligns with the high frequency of the *RNF213* p.Arg4810Lys variant in the East Asian populations. *RNF213* p.Arg4810Lys has been identified as a major susceptibility allele for MMD in East Asians [8]. In Korea, this variant has an allele frequency of approximately 1.36% in the general population [9–11], and the incidence of MMD has been reported as 1.7 to 2.3 per 100,000 persons [12]. Japanese individuals have reported a similar allele frequency of 1.36% in the general population [11]. Additionally, p.Arg4810Lys is associated with non-MMD intracranial artery stenosis or occlusion (ICASO), suggesting that its impact extends beyond classical MMD to broader cerebrovascular pathologies [13,14].

CADASIL is a small-vessel disease caused by pathogenic variants of *NOTCH3*, resulting in recurrent stroke, cognitive impairment, and psychiatric disturbances [3]. CADASIL was previously regarded as a rare genetic disorder, with a reported clinical prevalence of 2–5 per 100,000 individuals [15–17]. However, a recent study that leveraged population databases suggested a much higher global mutation prevalence. According to Rutten et al., the overall frequency of cysteine-altering *NOTCH3* variants is estimated at 3.4 per 1,000 individuals globally, with even higher frequencies reported in East (9.0/1,000) and South Asian (11.7/1,000) populations [18]. Several studies have reported ethnic variability in the distribution of *NOTCH3* pathogenic variants [19,20]. In East Asia, the frequency of pathogenic cysteine-altering variants is approximately 9 in 1,000 individuals, the second highest worldwide after South Asia [18]. Among Korean patients, p.Arg544Cys and p.Arg75Pro are most frequently observed [20]. While p.Arg544Cys follows the classical cysteine-altering pathogenic mechanism, p.Arg75Pro is a cysteine-sparing variant, expanding the genetic spectrum of CADASIL and suggesting that its true prevalence may be underestimated.

Although *RNF213* and *NOTCH3* variants show incomplete penetrance, carriers may have an elevated stroke risk when exposed to environmental factors such as smoking, hypercholesterolemia, and a sedentary lifestyle. This gene–environment interaction underscores the importance of early genetic screening in high-risk populations. In particular, in regions where stroke prevalence is high, such as East Asia, early identification of high-risk individuals can improve risk stratification and personalized management and ultimately reduce the burden of cerebrovascular disease. In this study, we analyzed the allele frequencies and estimated the genetic prevalence of *RNF213* and *NOTCH3* variants in over 120,000 healthy Korean individuals.

## Methods

### Study subjects

A total of 129,933 individuals from across South Korea, who voluntarily opted for genetic testing as part of their health checkups using the Genome Health test between July 2021 and August 2024, were included as study participants. These health examinations were conducted at more than 200 local clinics nationwide, primarily in urban areas. Genome Health is a single-nucleotide polymorphism genotyping test developed by GC Genome, which utilizes next-generation sequencing technology to identify risk alleles associated with various diseases, including cerebrovascular disorders. All DNA samples from the individuals were collected from unrelated Koreans, and the study protocol was reviewed and approved by the Institutional Review Boards of GC Labs (GCL-2024-1054-01). This study complied with the tenets of the Declaration of Helsinki for human subjects. Given the retrospective nature of the study and the use of anonymized data, the requirement for informed consent was waived. The data used in this study was accessed on October 15, 2024.

### Selection of variants related to cerebrovascular disorders

MMD-associated *RNF213* p.Arg4810Lys (rs112735431) and three CADASIL-associated *NOTCH3* variants, p.Arg544Cys (rs201118034), p.Arg640Cys (rs760768552), and p.Arg75Pro (rs145069047), were selected. *RNF213* p.Arg4810Lys is a major genetic risk factor for MMD in East Asians [9,10], found in 67% of Korean patients with MMD, and is associated with a high odds ratios of 48–63 [21]. The specific region of *RNF213* spanning 120 base pairs was located at chr17:78358884_78359004. Given that this study analyzed data from the general population, we selected three *NOTCH3* variants based on their allele frequencies in gnomAD v4.1.0 [22]. p.Arg544Cys and p.Arg640Cys were the two most frequent cysteine-altering pathogenic variants, with allele frequencies of 0.14% and 0.02%, respectively, in gnomAD East Asian. p.Arg75Pro was the most frequent cysteine-sparing pathogenic variant, with a 0.02% allele frequency in gnomAD East Asian. Three specific regions of *NOTCH3* spanning 120 base pairs each were located at chr19:15297661_15297781, chr19:15298065_15298185, and chr19:15303243_15303363. The reference sequences NM_001256071.3 and NM_000435.3 were used for *RNF213* and *NOTCH3*, respectively. Detailed information on each variant is provided in Table 1.

**Table 1:** Variants analyzed in this study.

Gene	Phenotype	Inheritance pattern	Nucleotide change	Protein change	rs number
*RNF213*	Moyamoya disease	AD, AR^a^	c.14429G > A	p.Arg4810Lys	rs112735431
*NOTCH3*	CADASIL	AD	c.1918C > T	p.Arg640Cys	rs760768552
			c.1630C > T	p.Arg544Cys	rs201118034
			c.224G > C	p.Arg75Pro	rs145069047

AD, autosomal dominant; AR, autosomal recessive; CADASIL, cerebral arteriopathy with subcortical infarcts and leukoencephalopathy. Reference sequences are NM_001256071.3 for *RNF213* and NM_000435.3 for *NOTCH3*.

^a^Autosomal dominant inheritance with incomplete penetrance. Early onset and severe cases have also been reported, suggesting an autosomal recessive inheritance.

### Genetic analysis

Genomic DNA was extracted from EDTA whole blood samples. A custom panel (IDT, Coralville, IA, USA) was used for library preparation, and sequencing was performed using the NovaSeq platform (Illumina, San Diego, CA, USA). DNA sequence reads were aligned to the reference sequence based on the public human genome build GRCh37/UCSC hg19. Alignment was performed with BWA-mem (version 0.7.17), duplicated reads were marked with biobambam2 base quality recalibration, variant calling was performed with the Genome Analysis Tool kit (version 4.1.8), and annotation was performed using a custom Python script.

### Comparison of the prevalence estimates using population databases

Prevalence estimates of risk alleles from *RNF213* and *NOTCH3* were determined using public population databases. We obtained population frequency data from gnomAD (v4.1.0), accessible at https://gnomad.broadinstitute.org/. The gnomAD database consists of 730,947 exomes and 76,215 genomes from diverse populations, including 30,019 from Admixed American, 37,545 from African/African American, 14,804 from Ashkenazi Jewish, 456 from Amish, 22,448 from East Asian, 32,026 from European (Finnish), 590,031 from European (non-Finnish), 3,031 from Middle Eastern, 45,546 from South Asian, and 31,256 from other genetic ancestries. For Korean-specific data, we used the KOVA database (v2), which includes information from 5,305 Koreans (accessible at https://www.kobic.re.kr/kova/) [23].

### Statistical analysis

Differences in allele frequencies among groups according to age and sex were analyzed using the chi-squared test. Bonferroni correction was used for multiple comparisons across age categories. To estimate genetic prevalence, MMD and CADASIL were defined by the presence of the analyzed variants, assuming an autosomal dominant inheritance pattern. Genetic prevalence was estimated using the Hardy-Weinberg equilibrium principle (1 = p² + 2pq + q²), where p represents the major allele (non-disease allele) and q represents the minor allele (disease allele). The observed allele frequencies of the analyzed variants (*RNF213* p.Arg4810Lys for MMD and *NOTCH3* p.Arg544Cys, p.Arg640Cys, and p.Arg75Pro for CADASIL) were used as q in the calculation. The estimated disease prevalence was predicted using 2pg + q². Calculations were performed using R version 4.3.2, and 95% confidence intervals (CI) were calculated for each value. All *P*-values were based on two-sided comparisons, and *P*-values < 0.05 were considered to indicate statistical significance.

## Results

### Baseline characteristics of study subjects

Between July 2021 and August 2024, 129,933 individuals undergoing health checkups were included in this study. The median age of the participants was 42 years (range 18–93 years). The age distribution of the population was as follows: 12.8% were under 30 years, 28.9% were in their 30s, 26.6% were in their 40s, 19.4% were in their 50s, and 12.3% were 60 years or older. Among the participants, 55% were male and 45% were female.

### Allele frequencies and prevalence estimates in MMD

*RNF213* p.Arg4810Lys showed an allele frequency of 1.08% (95% CI: 1.04–1.12%). A total of 13 homozygotes was identified, of which four were under 30 years of age and three were in their 30s, 40s, and 50s. No homozygotes were identified in the 60 years or older group. The estimated genetic prevalence of MMD, based on p.Arg4810Lys, was approximately 1 in 47 individuals (95% CI: 1/48–1/45) (Table 2). Allele frequencies were not significantly different according to age and sex (Table 3).

**Table 2:** Allele frequencies and prevalence estimates in this study.

Gene	Variant	Genotype			Allele frequency % (95% CI)	Prevalence estimate 1/N (95% CI)
		Allele count	Het	Hom		
*RNF213*	c.14429G>A; p.(Arg4810Lys)	2,808	2,782	13	1.08 (1.04–1.12)	1/47 (1/48–1/45)
*NOTCH3*	c.1918C>T; p.(Arg640Cys)	180	180	0	0.07 (0.06–0.08)	1/706 (1/805–1/655)
*NOTCH3*	c.1630C>T; p.(Arg544Cys)	185	181	2	0.07 (0.06–0.08)	1/703 (1/782–1/638)
*NOTCH3*	c.224G>C; p.(Arg75Pro)	104	104	0	0.04 (0.03–0.05)	1/1,250 (1/1,100–1/1,446)

Het, heterozygous; Hom, homozygous.

**Table 3:** Allele frequencies according to age and sex.

Variable	Subject number	*RNF213* p.Arg4810Lys		*NOTCH3* p.Arg640Cys		*NOTCH3* p.Arg544Cys		*NOTCH3* p.Arg75Pro	
		allele frequency % (95% CI)	P	allele frequency % (95% CI)	P	allele frequency % (95% CI)	P	allele frequency % (95% CI)	P
Age, yrs			0.976		0.025^a^		0.034^a^		0.477
<30	16,682	1.10 (0.99-1.22)		0.07 (0.04-0.11)		0.09 (0.06-0.13)		0.04 (0.02-0.07)	
30-39	37,516	1.07 (1.00-1.15)		0.08 (0.06-0.10)		0.09 (0.07-0.11)		0.04 (0.03-0.06)	
40-49	34,608	1.09 (1.02-1.17)		0.09 (0.07-0.11)		0.05 (0.03-0.07)		0.04 (0.03-0.06)	
50-59	25,150	1.06 (0.97-1.15)		0.05 (0.03-0.08)		0.07 (0.05-0.10)		0.04 (0.03-0.07)	
≥60	15,977	1.08 (0.97-1.22)		0.04 (0.02-0.07)		0.07 (0.05-0.11)		0.02 (0.01-0.05)	
Sex			0.223		0.282		0.087		0.019
Male	71,696	1.06 (1.01-1.11)		0.07 (0.06-0.09)		0.06 (0.05-0.08)		0.03 (0.02-0.04)	
Female	58,237	1.11 (1.05-1.17)		0.06 (0.05-0.08)		0.08 (0.07-0.10)		0.05 (0.04-0.07)	

^a^After applying Bonferroni correction for multiple comparisons, none of the pairwise differences between the age groups remained statistically significant. CI, confidence interval.

### Allele frequencies and prevalence estimates in CADASIL

For CADASIL, the allele frequencies of *NOTCH3* variants were 0.07% (95% CI: 0.06–0.08%) for p.Arg640Cys, 0.07% (95% CI: 0.06–0.08%) for p.Arg544Cys, and 0.04% (95% CI: 0.03–0.05%) for p.Arg75Pro (Table 2). Two individuals were homozygous for p.Arg544Cys. The estimated genetic prevalence of CADASIL, based on these three variants, was 1 in 277 individuals (95% CI: 1/261–1/296). Allele frequencies were not significantly different according to age but showed a significant difference according to sex (p = 0.019) (Table 3).

Additionally, eight individuals were identified to have both *RNF213* and *NOTCH3* variants: two with p.Arg640Cys, five with p.Arg544Cys, and one with p.Arg75Pro.

### Comparison of the prevalence estimates with the population databases

**Fig 1:**
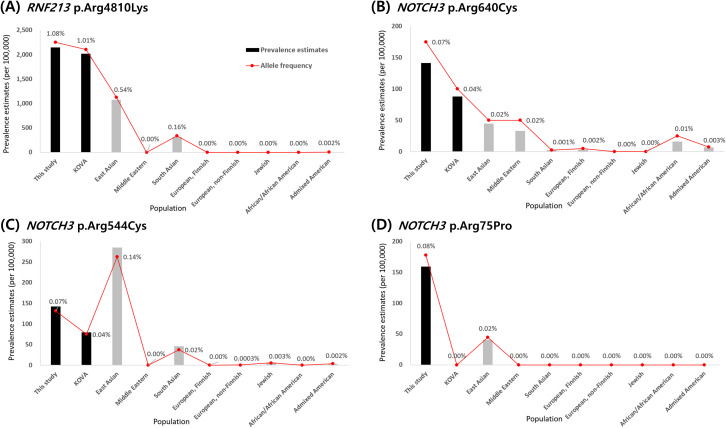
Comparison of the allele frequencies (A) and estimated genetic prevalence per 100,000 individuals (B) across populations. Allele frequencies and prevalence estimates for the global population were calculated using the gnomAD (v4.1.0) database.

Allele frequencies and prevalence estimates were compared with public databases (Fig 1). The allele frequency of *RNF213* p.Arg4810Lys in this study was 1.08%, which was comparable to that reported in KOVA (1.01%) but approximately twice as high as that reported in the gnomAD East Asian (0.54%). This variant was almost absent in non-East Asian populations except South Asians, showing 0.16% allele frequency.

Among the *NOTCH3* variants, both p.Arg640Cys and p.Arg75Pro showed higher allele frequencies in this study compared to gnomAD East Asian and KOVA. On the other hand, p.Arg544Cys showed an allele frequency of 0.07%, which was approximately half of that reported in gnomAD East Asian (0.14%) but was more prevalent than that reported in KOVA (0.04%).

## Discussion

This study represents the largest population-based analysis of *RNF213* and *NOTCH3* variants in Koreans and provides valuable insights into the genetic epidemiology of cerebrovascular disorders in Koreans. These findings reinforce the high genetic burden of MMD and CADASIL in Koreans and highlight the importance of population-specific genetic screening for improving stroke management.

The allele frequency of 1.08% for *RNF213* p.Arg4810Lys in this study was consistent with previous Korean studies [9,11]. Notably, this frequency was approximately twice that of gnomAD East Asian (0.54%), suggesting subpopulation differences even within East Asians. Previous studies have reported allele frequencies of approximately 1.36% in the Japanese and 0.43% in Chinese populations, further supporting subpopulation differences within East Asia. This discrepancy suggests that the aggregated gnomAD East Asian frequency may underrepresent the true genetic risk in subpopulations and underscores the importance of large-scale population-specific genetic studies.

Although *RNF213* p.Arg4810Lys is frequently observed in East Asia, its penetrance was reported to be 1/150–1/300 [24]. Therefore, the clinical prevalence of MMD was estimated to be between 1 in 7,050 individuals and 1 in 14,100 individuals. However, recent studies have implicated *RNF213* p.Arg4810Lys in ICASO [13,14], suggesting that even if the variant does not always lead to classical MMD, its presence may contribute to other cerebrovascular disorders. Indeed, according to a previous study of healthy relatives of MMD patients, 23.5% of p.Arg4810Lys carriers developed ICASO [25]. Therefore, determining the population frequency of *RNF213* p.Arg4810Lys is critical not only for understanding MMD risk, but also for effective risk stratification and management of ICASO. The findings of this study provide essential data for integrating genetic screening with routine cerebrovascular disease risk assessments in East Asia.

Homozygosity for *RNF213* p.Arg4810Lys is associated with an earlier onset and more severe phenotype of MMD [5,6]. While heterozygous carriers exhibit low penetrance, the penetrance in homozygous individuals has been reported to exceed 78% [5]. Although rare, homozygous individuals have been reported in healthy populations. In a healthy Japanese population, the frequency of homozygotes was approximately 0.07% [26]. In the present study, 13 homozygotes were identified across a broader age range, including those in their 30s, 40s, and 50s, corresponding to a frequency of 0.01%. Further longitudinal studies are needed to characterize the phenotypic spectrum and long-term outcomes of *RNF213* homozygosity.

CADASIL is caused by pathogenic variants in *NOTCH3*, and the distribution of pathogenic variants varies significantly across populations. Caucasian, European, and Japanese patients have shown that the majority of *NOTCH3* pathogenic variants tend to cluster within exon 4, with commonly reported variants including p.Arg133Cys, p.Arg141Cys, p.Arg182Cys, and p.Arg169Cys. [19,27–30]. In contrast, in Korean and Chinese patients, a significant proportion of pathogenic variants tend to cluster in exon 11, with p.Arg544Cys being the most common [19,20]. A comparison with the gnomAD database supports these ethnic differences between the Korean and aggregated East Asian populations. p.Arg544Cys was more prevalent in the gnomAD East Asian population (0.14%) than that in the present study (0.07%). In contrast, p.Arg640Cys and p.Arg75Pro were more prevalent in this study than in gnomAD East Asian.

The estimated genetic prevalence of MMD and CADASIL in this study were 1/47 and 1/277, respectively. These findings suggest that a substantial proportion of the Korean population is genetically predisposed to stroke. Although genetic factors may have a limited effect when considered alone due to incomplete penetrance, their interaction with environmental factors can elevate stroke risk among carriers. Therefore, the identification of carriers is important for personalized prevention strategies for modifiable environmental factors. Given the high prevalence of these genetic risk factors in Korean, early and targeted genetic screening could be helpful in identifying individuals at high risk for cerebrovascular disorders and facilitating personalized management, which can eventually reduce the stroke burden in Korea.

CADASIL is classically characterized as a small vessel disease. However, several studies in East Asians have reported atypical presentations, including large artery involvement [31–34]. One possible explanation for this phenomenon is the higher frequency of the *RNF213* p.Arg4810Lys in East Asians, which predisposes individuals not only to MMD but also to ICASO. Consequently, the co-occurrence of *RNF213* and *NOTCH3* variants may contribute to a broader spectrum of cerebrovascular pathologies in this population. The findings of this study, which demonstrate a high prevalence of *RNF213* p.Arg4810Lys in Koreans and its co-occurrence with *NOTCH3* variants, underscores the need to consider oligogenic interactions when assessing cerebrovascular risk in East Asians. Although the clinical significance of the eight individuals carrying both variants could not be assessed due to the absence of clinical and imaging data in this study, the observed co-occurrence highlights the importance of investigating potential synergistic effects between these genes. Further studies incorporating phenotypic data are required to elucidate the interactions between these genetic factors and to refine targeted screening and management strategies.

A key strength of this study is the large sample size of 129,933 healthy Korean individuals, which surpasses the sample sizes in public databases, such as gnomAD East Asian and KOVA. This extensive population study enabled us to derive robust and precise estimates of allele frequencies and genetic prevalence. This is important for developing effective population-specific screening and intervention strategies. Especially, in a region where stroke incidence is high, these genetic epidemiologic findings can lead to better-tailored interventions that address both genetic and modifiable environmental risk factors.

This study has some limitations. First, the genotype-phenotype correlation could not be assessed because of the absence of detailed clinical and imaging data. As the study population consisted of individuals undergoing health checkups rather than clinically diagnosed patients, access to comprehensive phenotypic information was inherently limited. Despite this limitation, the findings of this study provide a valuable foundation for future research. Combined with longitudinal clinical studies, our population-based data may contribute to more accurate estimations of disease burden and support risk stratification strategies tailored to East Asian populations. Second, this study only focused on one *RNF213* variant and three *NOTCH3* variants, and other pathogenic variants within these genes were not included in the analysis. This targeted approach may lead to underestimation of the full spectrum of genetic diversity associated with MMD and CADASIL. Further studies incorporating comprehensive variant analysis of *RNF213* and *NOTCH3* are needed to estimate the genetic prevalence of MMD and CADASIL more accurately. Lastly, because most participants were recruited through institutions located in urban areas, certain regional subpopulations may be underrepresented.

In conclusion, this study provides comprehensive genetic epidemiological data on MMD and CADASIL in a large Korean population. The high genetic prevalence of MMD and CADASIL may contribute to the high stroke burden in East Asia, when combined with environmental risk factors. These findings emphasize the importance of population-specific genetic studies for personalized cerebrovascular disease prevention and management.
